# Immunohistochemical Expression of Vitamin D Receptor (VDR) in Urinary Bladder Squamous Cell Carcinoma

**DOI:** 10.5146/tjpath.2023.12863

**Published:** 2024-05-18

**Authors:** Rehab Mohamed Sharaf, Passant Essam Eldin Shibel, Nadia Ahmed Abd El-Moeze

**Affiliations:** Department of Pathology, Beni-Suef University, Faculty of Medicine, Beni-Suef, Egypt; Cairo University, Faculty of Medicine, Cairo, Egypt

**Keywords:** VDR, Urinary bladder, Squamous cell carcinoma, Immunohistochemistry

## Abstract

***Objective: *** Squamous cell carcinoma (SCC) of the urinary bladder is associated with aggressive behavior and is typically treated with radical cystectomy. Vitamin D receptor (VDR) and its ligand Calcitriol have shown anti-tumor effects in various malignancies but to our knowledge there is no current information on VDR expression in bladder SCC. This study aimed to assess VDR immunostaining patterns in pure bladder SCC and its relation to the available clinicopathological parameters of such tumors.

***Material and Methods:*** VDR immunostaining was performed on 35 radical cystectomy specimens from patients with primary pure SCC. Nuclear and cytoplasmic VDR staining was scored separately using the semi-quantitative immunoreactive score.

***Results: *** Nuclear and cytoplasmic/membranous VDR expression was present in 35 (100%) and 19 (54.3%) cases, respectively, with a significant negative linear relationship (r=-0.33; p=0.035). Differences in cytoplasmic/membranous VDR expression were found in relation to tumor histology (p=0.018), tumor necrosis (p=0.022), and stage groups (p=0.001). Low cytoplasmic VDR correlated with increased tumor staging (Cc = -0.422), positive lymph node status (Cc = -0.375), and higher stage groups (Cc= -0.438). The median nuclear VDR expression score was significantly higher in advanced stage groups (p= 0.038).

***Conclusion:*** Our data suggest that VDR may be a potential prognostic factor in bladder SCC. Further studies and clinical trials using vitamin D supplements may provide a new therapeutic option for those high-risk patients.

## INTRODUCTION

According to GLOBOCAN data of 2020, bladder cancer is the 10th most frequently diagnosed cancer worldwide, with higher incidence and mortality rates among men (1).

Primary pure squamous cell carcinoma (SCC) is a rare histologic variant of bladder cancer, accounting for 2-5% of all bladder cancer cases. Unfortunately, it is associated with more aggressive behavior and is often diagnosed at a later stage compared to the most common urothelial carcinoma (2).

There are two subtypes of bladder SCC, bilharzial-associated and non-bilharzial-associated. Both are traditionally treated with radical cystectomy (3) without neoadjuvant chemotherapy because SCC, unlike urothelial carcinoma, is unresponsive to cisplatin-based chemotherapy (4). Therefore, tumor biomarkers and new therapeutic targets for bladder SCC must be investigated to improve survival outcomes.

The active metabolite of Vitamin D (1,25(OH)2-VitD3 or Calcitriol) affects many cellular functions (5) through binding to the Vitamin D receptor (VDR), a transcriptional regulatory factor that is primarily localized to the nucleus of target cells (6). Upon ligand (Calcitriol) binding, VDR forms a heterodimer with the retinoic X receptor. The heterodimer accumulates in the cell nucleus, where it regulates gene transcription via binding to vitamin D response elements (VDRE) in the promoter region of target genes. Transcriptional regulation can be either negative or positive depending on the specific VDRE sequences and the recruitment of co-activators or co-repressors (7,8).

Additionally, rapid non-genomic actions of 1,25(OH)2-VitD3 are mediated by plasma membrane-associated VDR. These actions include activation of several signaling pathways such as phosphatidylinositol-3’-kinase, phospholipase C, protein kinase C, and ion channels (9).

Calcitriol and its receptor VDR have antitumor effects as demonstrated by many experimental studies in different types of malignant tumors including bladder urothelial carcinoma (10,11). In tumor cells, Vitamin D is shown to induce apoptosis and cell differentiation. Also, it inhibits cellular proliferation, angiogenesis, invasion, and metastasis (12) suggesting that active vitamin D3 and its analogues may reduce the risk of cancer development and/or serve as a potential therapeutic agent.

In the literature, several studies have investigated the role of VDR expression in SCC of different organs such as the oral cavity (13), esophagus (14), lung (15), and vulva (16). However, the information on VDR expression in bladder SCC is significantly lacking. Therefore, we aimed to analyze the VDR immunostaining pattern in patients with bladder SCC undergoing radical cystectomy and study its relation to the available clinicopathological parameters.

## MATERIALS and METHODS

### Case Selection

Radical cystectomy specimens from patients with primary pure bladder SCC (n=37) were retrospectively selected from the pathology lab of a specialized medical center from January 2019 to December 2022. Formalin-fixed and paraffin-embedded (FFPE) tumor tissue blocks of the selected cases were retrieved from the archives. Two patients with unavailable tumor tissue blocks were excluded from further analysis. No patients had metastatic disease or received neoadjuvant therapy prior to surgery.

The available clinical and pathological data of all patients (n=35) including age, sex, tumor stage, and nodal status were obtained from the pathology requests and reports. Patients’ names were removed and changed to numbers. This study was conducted after institutional ethical committee approval (FMBSUREC/02052023).

### Histopathology

Tissue sections were cut from each FFPE block using a rotatory microtome at 4-5 µm thickness and were stained with Hematoxylin and Eosin stain. Slides were reviewed to confirm tumor type, and grading was based on WHO classification of urinary and male genital tumors, 5th edition (17). The slides were also examined for the presence of vascular invasion, perineural invasion, bilharzial ova, and necrosis. Staging was performed according to the TNM system elaborated by the American Joint Committee on Cancer (AJCC) and the Union for International Cancer Control (UICC) (18).

### VDR Immunohistochemistry (IHC)

Immunohistochemical staining was performed using anti-VDR antibody clone D6 (mouse monoclonal, diluted 1:200, Catalog number #MC0304RTU7, Medaysis, San Francisco Bay Area, USA). Sections 4 μm thick were dewaxed in xylene, dehydrated by an alcohol gradient, and then subjected to microwave heating for antigen retrieval. After incubation with the primary antibody for an hour at room temperature, sections were treated with the biotin-conjugated secondary antibody followed by the streptavidin biotin peroxidase complex. The reactions became visible after adding 3,3’-diaminobenzidine tetrahydrochloride. Slides were then counterstained in hematoxylin, dehydrated, and mounted. Negative controls were similarly prepared except for adding phosphate-buffered saline instead of the primary antibody. For positive control, skin tissue was used in each staining session.

### VDR Immunohistochemical Evaluation

Positive Immunoreactivity was detected as brownish granules in the cell membrane/cytoplasm or nucleus. VDR expression was evaluated using the semi-quantitative immunoreactive score (IRS); the product of multiplying the scores for the percentage of positive cells and the staining intensity.

The percentage of cells expressing VDR in relation to the total number of tumor cells was scored as: 0 (<10% positive cells), 1 (11–30% positive cells), 2 (31–75% positive cells), and 3 (>75% positive cells). For staining intensity, a score of 0, 1, 2 and 3 was given for negative, weak, moderate, and strong brown staining, respectively. The final IRS was performed separately for cytoplasmic/membranous and nuclear expression in each tumor sample and the values ranged from 0 to 9. We then categorized VDR expression into negative (scores of 0 to 1), low (scores of 2 to 4), and high (scores of 6 to 9) (19).

Slides were examined under light microscopy (Olympus model BX53) by two independent authors who were blinded to all data. Images were obtained using a microscope slide scanner (APERIO LV1, Leica Biosystems).

### Statistical Analysis

The statistical analysis was performed using IBM SPSS for Windows version 25 (Armonk, New York: IBM Corp.), with a significant difference at *p*-value less than 0.05. Categorical variables were described in form of frequencies and percentages, whereas non-parametric continuous variables were described as the median (IQR). Data normality was tested using the Shapiro-Wilk Test of Normality, and then the Mann-Whitney U test was used to analyze the differences between two unrelated groups in terms of continuous variables, The Kruskal-Wallis test for (k)-unrelated groups and the Wilcoxon Signed Rank Test was used to analyze the differences between two related samples in terms of continuous variables (VDR in cytoplasmic vs. nuclear expression). For categorical variables, the Chi-square (χ2) and Fisher’s exact (when more than 20% of cells have expected frequencies <5) tests were used. The cytoplasmic and nuclear expression scores were correlated with each other as well as with other variables using the Pearson correlation coefficient and Spearman’s rank correlation, respectively, with correlation coefficient (r) values ranging from −1 to +1.

## RESULTS

### Demographic and tumoral features

Out of the 35 patients, 27 were males with a 3.4:1 male to female ratio and median age of 62 (range: 52-78) years. Tumor size ranged from 4 to 8 cm (median: 5.5 cm). Most tumors were of moderate to high grade and the keratinizing-type (KSCC) was more prevalent (57.1%). Muscle invasion was detected among most of the studied tumors (91.4%). Sixty percent of patients presented with stage group IIIA-B. Further patient characteristics are listed in detail in Table 1.

**Table 1:** Clinicopathological characteristics of the bladder squamous cell carcinoma patients.

**Characteristics**		**Frequency**	**Percent**
Sex	Male	27	77.1
	Female	8	22.9
Age	≤ 60	15	42.9
	> 60	20	57.1
Tumor histology	KSCC	20	57.1
	NKSCC	15	42.9
Grade	GI	3	8.6
	GII	22	62.9
	GIII	10	28.6
Muscle invasion	Absent	3	8.6
	present	32	91.4
Tumor stage	T1	3	8.6
	T2	11	31.4
	T3	17	48.6
	T4	4	11.4
Bilharziasis	Absent	16	45.7
	Present	19	54.3
Necrosis	Absent	14	40.0
	Present	21	60.0
Lymphovascular invasion	Negative	25	71.4
	Positive	10	28.6
Perineural invasion	Negative	24	68.6
	Positive	11	31.4
Lymph node metastasis	Negative	25	71.4
	Positive	10	28.6
Nodal stage	N0	25	71.4
	N1	7	20.0
	N2	3	8.6
Stage group	I	3	8.6
	II	11	31.4
	IIIA	18	51.4
	IIIB	3	8.6

**KSCC:** Keratinizing squamous cell carcinoma, **NKSCC:** Non-keratinizing squamous cell carcinoma

### Assessment of VDR expression

**Figure 1:**
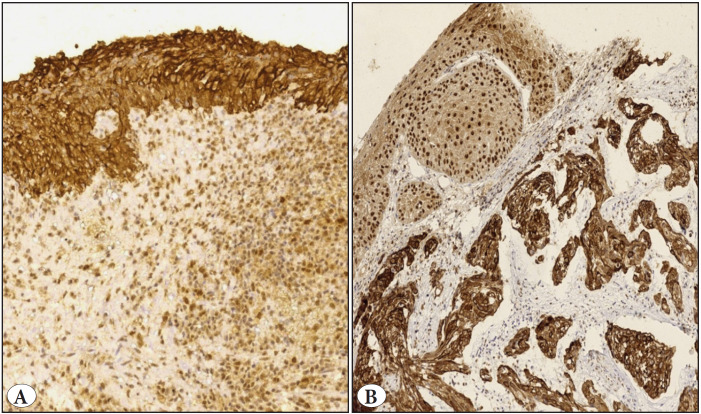
VDR immunohistochemistry of apparently normal urothelium **(A)** shows diffuse strong cytoplasmic/membranous staining (Original magnification x200). **B)** VDR nuclear expression in metaplastic squamous epithelium, note cytoplasmic immunostaining in the underlying malignant squamous cells (Original magnification x100).

In this study, VDR expression was predominantly located in the cytoplasm and the cell membrane of the histologically normal urothelium, which was included in 10 cases (10/10). In contrast, areas of squamous metaplasia of the covering non-neoplastic urothelium seen in 15 cases showed only nuclear staining for VDR (15/15) Figure 1.

**Figure 2:**
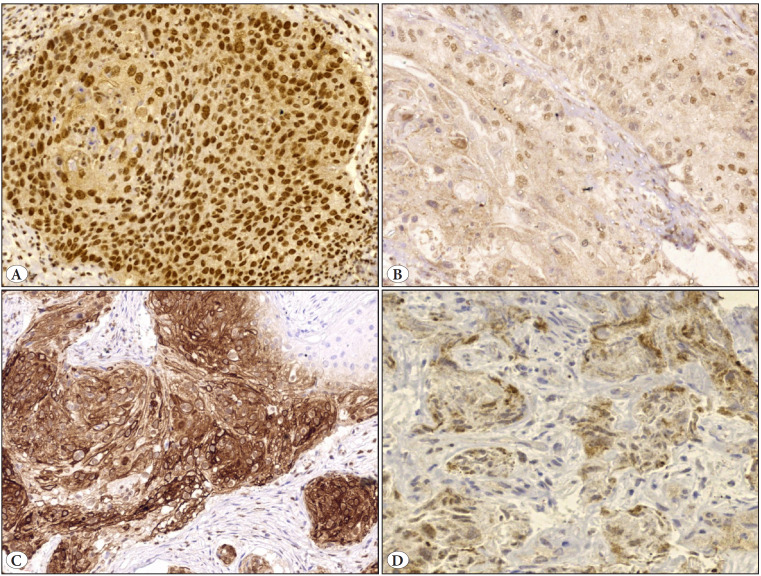
VDR immunostaining of bladder squamous cell carcinoma: **A)** Strong and diffuse nuclear staining. **B)** Weak nuclear staining. **C)** Strong cytoplasmic/membranous positivity. **D)** Low cytoplasmic expression (Original magnification A, B, C x200, D x400).

**Figure 3:**
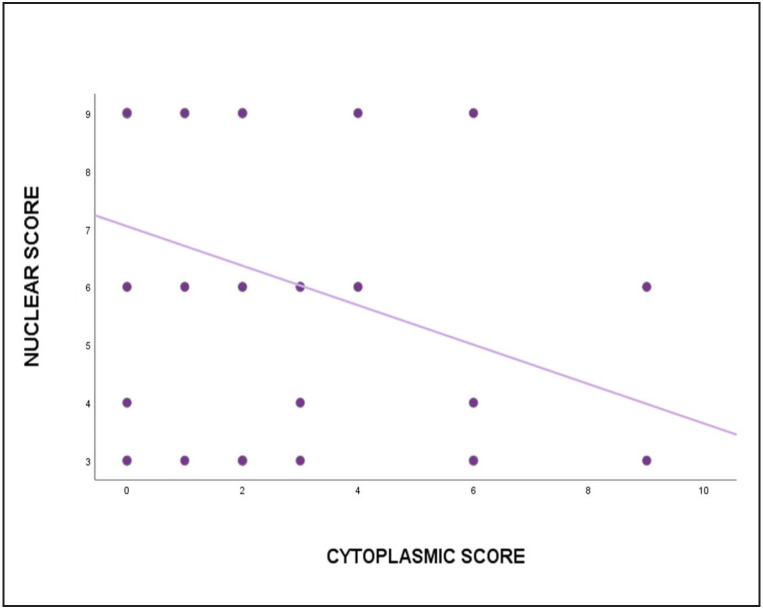
Scatter plot showing the relationship between nuclear and cytoplasmic VDR expression scores.

All tumors showed low or high nuclear immunostaining with no recorded negative cases. Cytoplasmic/membranous VDR expression was detected in tumor cells of 19 (54.3%) cases, and of these 37.1% showed low expression and 17.1% showed high expression. The median nuclear VDR expression was significantly (p<0.001) higher in bladder SCC as compared to the cytoplasmic/membranous VDR Table 2, Figure 2 with a significant negative linear correlation (r=-0.33; p=0.035) Figure 3.

**Table 2:** VDR expression scores in the 35 studied cases.

**Immunoreactive score (IRS)**		**VDR expression**	
		**Cytoplasmic/ membranous**	**Nuclear**
Negative	0	11 (31.4%)	-
	1	5 (14.3%)	-
Low	2	7 (20%)	-
	3	4 (11.4%)	10 (28.6%)
	4	2 (5.7%)	3 (8.6%)
High	6	4 (11.4%)	7 (20%)
	9	2 (5.7%)	15 (42.9%)
Median (IQR)		2 (3)	6 (6)
P value		<0.001*	

**IQR:** Interquartile range, *Statistical analysis by non-parametric Wilcoxon Signed Ranks Test for related samples (nuclear vs. cytoplasmic scores)

A high cytoplasmic VDR level was found in non-keratinizing tumors (p=0.018) and in those with absent necrosis (p=0.022). Cytoplasmic IRS significantly decreased with progressing AJCC stage groups (p=0.001). It was also lower in cases with positive lymph node metastasis (p=0.051). In contrast, nuclear IRS significantly increased in patients with advanced stage groups (p=0.038) Table 3.

**Table 3:** Distribution of bladder SCC by nuclear and cytoplasmic VDR expression.

**Characteristics**		**n= 35**	**Cytoplasmic IRS**			**Nuclear IRS**		
			**Median**	**IQR**	**p-value**	**Median**	**IQR**	**p-value**
Sex(a)	Male	27	2.00	3.00	0.697	6.00	6.00	0.888
	Female	8	2.00	3.50		7.50	6.00	
Tumor histology(a)	KSCC	20	1.00	2.00	0.018*	6.00	6.00	0.884
	NKSCC	15	3.00	6.00		6.00	6.00	
Grade(b)	GI	3	4.00	-	0.836	9.00	-	0.345
	GII	22	2.00	3.00		6.00	5.25	
	GIII	10	1.50	3.75		3.50	6.00	
Muscle invasion(a)	Absent	3	1.00	-	0.989	9.00	0.00	0.057
	present	32	2.00	3.00		6.00	6.00	
Bilharziasis(a)	Absent	16	1.00	3.75	0.51	5.00	6.00	0.603
	present	19	2.00	3.00		6.00	6.00	
Necrosis(a)	Absent	14	2.00	5.25	0.022*	6.00	5.25	0.662
	present	21	1.00	3.00		6.00	6.00	
LV-invasion(a)	Negative	25	2.00	3.50	0.65	6.00	5.50	0.952
	Positive	10	1.00	3.00		7.50	6.00	
Perineural invasion(a)	Negative	24	2.00	3.00	0.292	6.00	5.75	0.804
	Positive	11	0.00	3.00		6.00	6.00	
LN metastasis(a)	Negative	25	2.00	4.00	0.051	6.00	6.00	0.076
	Positive	10	0.00	2.25		9.00	3.75	
Stage group(a)	(I - II)	14	3.50	5.00	0.001*	4.00	3.75	0.038*
	(IIIA-IIIB)	21	1.00	2.00		9.00	4.50	

**IRS:** Immunoreactive score, **IQR:** Interquartile range, **KSCC:** keratinizing squamous cell carcinoma, **LN:** lymph node, **LV: l**ymphovascular, **NKSCC:** non-keratinizing squamous cell carcinoma -Statistical analysis was conducted by (a) Mann-Whitney test, (b) Kruskal-Wallis one-way ANOVA test, *statistically significant difference (p <0.05)

The cytoplasmic VDR expression score showed a statistically significant negative moderate correlation with T-stage, nodal status, and stage groups by Spearman’s analysis. However, the nuclear expression score did not correlate with the clinicopathological data Table 4.

**Table 4:** Correlation between cytoplasmic, nuclear VDR expression and clinicopathological variables.

**Variables**	**Cytoplasmic VDR expression**		**Nuclear VDR expression**	
	**p**	Correlation coefficient*	**p**	**Correlation coefficient**
Age	0.270	.192	0.943	0.012
Tumor size	0.684	-0.071	0.748	-0.056
Tumor stage	0.012	-0.422**	0.115	0.271
Nodal stage	0.027	-0.375**	0.061	0.320
Stage groups	0.009	-0.438***	0.095	0.287

*Spearman’s correlation analysis, ** Correlation is significant at the 0.05 level, *** Correlation is significant at the 0.01 level, p two-tailed significance

## DISCUSSION

Generally, urinary tract infection, Schistosomiasis, and other bladder irritants lead to chronic inflammation that is a major contributor to carcinogenesis of bladder SCC (17). Growth factors and cytokines released in chronic inflammation promote cell proliferation, migration, and angiogenesis, and inhibit apoptosis, resulting in squamous metaplasia, dysplasia, and invasive carcinoma (20).

Calcitriol has shown anti-inflammatory activities through inhibition of prostaglandin action, down regulation of p38 stress kinase, and nuclear factor KB signaling (21). Also, Calcitriol strongly inhibits cell proliferation in many human cancers including squamous cell carcinoma. The anti-proliferative effect depends on the levels of VDR (22) where mutations or deletion of VDR and the hydroxylase enzyme (CYP27B1) result in increased cell proliferation within the epidermal basal layer as well as defects in permeability barrier formation and the innate immune response (23).

In a clinical study, Calcitriol supplementation for 3 weeks before the surgical procedure in patients with head and neck SCC has prolonged the recurrence time (24). This highlights the role of Vitamin D as a potential novel, adjuvant cancer therapy with minimal side effects. To our knowledge, this study is the first to describe VDR expression exclusively in primary pure SCC of the urinary bladder.

Our results showed that there was more nuclear than cytoplasmic expression in all tumor samples with significant statistical difference. Similar findings were reported for different grades of cutaneous SCC (25), lung SCC (15,26), vulvar squamous carcinoma (16), and oral SCC (19). In contrast, Peng et al. found that VDR expression in esophageal SCC was predominantly localized to the cytoplasm and the cell membrane (14). In this study, increased nuclear VDR expression was accompanied by a decrease in cytoplasmic expression, displaying a significant inverse correlation. Yet, Czogalla et al. observed no correlation between nuclear and cytoplasmic VDR expression in epithelial ovarian carcinomas (27). It is worthy to mention that such a relationship is sparsely reported in the literature and may require further studies for confirmation.

Moreover, Nuclear VDR expression was observed in the metaplastic squamous epithelium seen near some tumors. This finding is compatible with the result reported by Menezes et al. for metaplastic lung biopsies (26).

Increased nuclear VDR expression in bladder SCC may be a sign for genomic pathways of vitamin D that control the abnormal growth, either metaplastic or neoplastic. However, cytoplasmic VDR may represent an adaptive mechanism for malignant cells. This may also point to increased sensitivity of tumor tissue to vitamin D activity.

Although nuclear VDR was predominant in patients’ tumor tissues, it showed only a significant statistical relationship with the AJCC stage groups (nuclear expression increased in more advanced tumors). This finding is in contrast to the result reported by Anand et al. where IRS significantly decreased with progressing anatomic stages in oral SCC cases (19). Srinivasan et al. and Del Puerto et al. found no significant correlation between nuclear VDR positivity and AJCC staging in lung cancer and cutaneous melanoma, respectively (15,28). We also observed a trend towards an increased expression of nuclear VDR in cases with metastatic lymph nodes versus those without nodal deposits. However, the difference was not statistically significant (p=0.076).

In the present study, the cytoplasmic VDR level was found to be significantly lower in keratinizing-type SCCs and necrotizing tumors. When correlated with the AJCC anatomic stage/prognostic groups, cytoplasmic expression significantly decreased across the stages, indicating loss of VDR expression with increased tumor progression. Although statistically insignificant, a trend for decreased cytoplasmic VDR levels in tumors with poor differentiation, positive lymphovascular invasion, and perineural invasion was found.

It is difficult to compare the above results because no other studies on VDR expression in bladder SCC have been conducted yet. However, our findings are in line with previous studies on various types of cancers like esophageal carcinoma (14), cutaneous melanoma (28), and colorectal cancer (29). They reported that loss of cytoplasmic VDR expression is associated with differentiation and TNM stage. Different results were found by Czogalla et al. where high cytoplasmic VDR staining positively correlated with lymph node metastasis as well as a higher FIGO stage in cases of ovarian cancer (27). Others showed no association between cytoplasmic VDR and clinicopathological variables or the overall survival (15,16).

This discrepancy between studies can be explained by the different studied tumor types, different used VDR antibody clones, variable cut offs used for positivity, as well as polymorphisms in the VDR gene. However, the negative correlation between cytoplasmic VDR expression and tumor progression in some cancer types suggests VDR as a potential target of downregulation or ablation.

The small number of cases with pure bladder SCC seen in our center and the absence of their follow up data are the main limitations of our work.

## CONCLUSION

The current study shows that VDR expression is present in histologically normal urothelium, squamous metaplasia, and invasive bladder SCC. Nuclear VDR expression is significantly higher in tumor samples than cytoplasmic VDR. Nuclear expression significantly increased in advanced tumor stages. Cytoplasmic VDR staining is inversely correlated with tumor staging, nodal status, and the AJCC stage groups, suggesting that loss of cytoplasmic VDR may be a prognostic factor for bladder SCC. Future large studies including clinical trials are recommended to prove our hypothesis that it may positively influence the patient management.

## Conflict of Interest

The authors declare no conflicts of interest.

## Funding

No funding has been received.

## Ethics Approval

This study was conducted after Beni-Suef University ethical committee approval (FMBSUREC/02052023).
